# Severe atopic dermatitis treated with anti-interleukin 4Rα reduces the psychological burden in patients with and without alexithymia

**DOI:** 10.1016/j.jdin.2023.07.020

**Published:** 2023-08-31

**Authors:** Vincenzo Patella, Eustachio Nettis, Tiziana Peduto, Luciana Pierro, Giovanni Pellacani, Laura Bonzano, Maria Stefania Leto Barone, Simona Palmieri, Roberta Zunno

**Affiliations:** a Department of Internal Medicine ASL Salerno, “Santa Maria della Speranza” Hospital, Salerno, Italy; b Postgraduate Program in Allergy and Clinical Immunology, University of Naples “Federico II”, Naples, Italy; c Department of Emergency and Organ Transplantation, School and Chair of Allergology and Clinical Immunology, University of Bari “Aldo Moro”, Bari, Italy; d Dermatology Clinic, Department of Clinical Internal, Anesthesiological and Cardiovascular Sciences, University of Rome “Sapienza”, Rome, Italy; e Dermatology Unit, Surgical, Medical and Dental Department of Morphological Sciences related to Transplant, Oncology and Regenerative Medicine, University of Modena and Reggio Emilia, Modena, Italy; f Functional Unit Internal Medicine “Casa di Cure Orestano”, Palermo, Italy; g Department of Mental Health, ASL Salerno, Salerno, Italy

**Keywords:** alexithymia, anti–IL-4Rα, anxiety, atopic dermatitis, depression, stress

*To the Editor:* Studies revealed a greater prevalence of clinically significant alexithymia in patients with atopic dermatitis (AD) than in controls, up to 66.7%.^1^^,^^2^ Alexithymia is a psychoaffective dysfunction characterized by the inability to identify, describe, and express feelings, restricted imagination, paucity of fantasy, dream construction, and concrete, logical, and realistic thinking.^3^ Data suggest that disease severity may predict alexithymia severity: for every one-point increase in Eczema Area and Severity Index (EASI), patients had a 9% enhanced likelihood of having a higher Toronto Alexithymia Scale-20 item total score and an 11% increase in likelihood of having a clinically significant Toronto Alexithymia Scale-20 item total score.^2^ In particular, a study found that alexithymia was observed in approximately 4 times more patients with AD than in the control group, and it was significantly associated with disease severity, but above all it was related to the intensity of itching.^4^ Our study evaluated the relation between alexithymia and the efficacy of anti-interleukin (IL) 4Rα in reducing the psychological burden in patients with AD, in real life. We interviewed 100 patients (age, 18-83 years, mean 36.2 ± 14.1; 54 men and 46 women) with AD and treated with anti–IL-4Rα. At beginning of treatment and after 16 weeks, clinical, quality of life (QoL), and psychological disorder scores were evaluated. The prevalence of alexithymia was also evaluated. Our results, in line with the data in the literature, show a high prevalence (78%) of alexithymia or indeterminate alexithymia in patients with AD. However, in our study, the presence of alexithymia did not affect the effectiveness of treatment with anti–IL-4Rα. Infact, in both patients with alexithymia and without alexithymia, treatment with anti–IL-4Rα improves psychological burden in patients with AD. Furthermore, after the treatment, EASI and Pruritus Numerical Rating Scale for pruritus improved more in patients with alexithymia than in patients without alexithymia, in a statistically significant way. This also applies to the Dermatology Quality of Life Index (Table I). In particular, results show a negative correlation between alexithymia and intensity of itching at T1: in patients with alexithymia, the symptoms of itching are statistically significantly reduced to a greater extent than in patients without alexithymia (Fig 1).

**Table I:** Differences in the response to the treatment between nonalexithymic and alexithymic subjects with repeated measures analysis of variance

SCORES		Patients without alexithymia, mean ± SD	Patients with alexithymia, mean ± SD	*P* value
EASI	T0	23.50 ± 13.49	30.85 ± 8.78	.005∗
	T1	5.43 ± 5.58	5.57 ± 3.31	
DLQI	T0	15 ± 7.4	17.46 ± 5.1	.004∗
	T1	6.05 ± 6.88	4.45 ± 3.24	
NRSP	T0	8.15 ± 1.6	8.27 ± 1.83	.014∗
	T1	3.25 ± 2.36	2.07 ± 1.54	
PSS	T0	20.94 ± 8.8	21.94 ± 7.97	.320
	T1	14.33 ± 5.34	13.48 ± 4.76	
HADS-A	T0	10.28 ± 5.99	10.76 ± 5.25	.236
	T1	5.56 ± 3.03	4.80 ± 3.39	
HADS-D	T0	8.83 ± 5.59	10.8 ± 4.53	.051
	T1	3.94 ± 2.7	3.73 ± 2.65	

*EASI*, Eczema Area and Severity Index; *DLQI*, Dermatology Life Quality Index; *NRSP*, Pruritus Numerical Rating Scale for pruritus; *PSS*, Perceived Stress Scale; *HADS-A and HADS-D*, Hospital anxiety (-A) and depression (-D) scale.

∗ *P* values indicate statistical significance.

**Fig 1:**
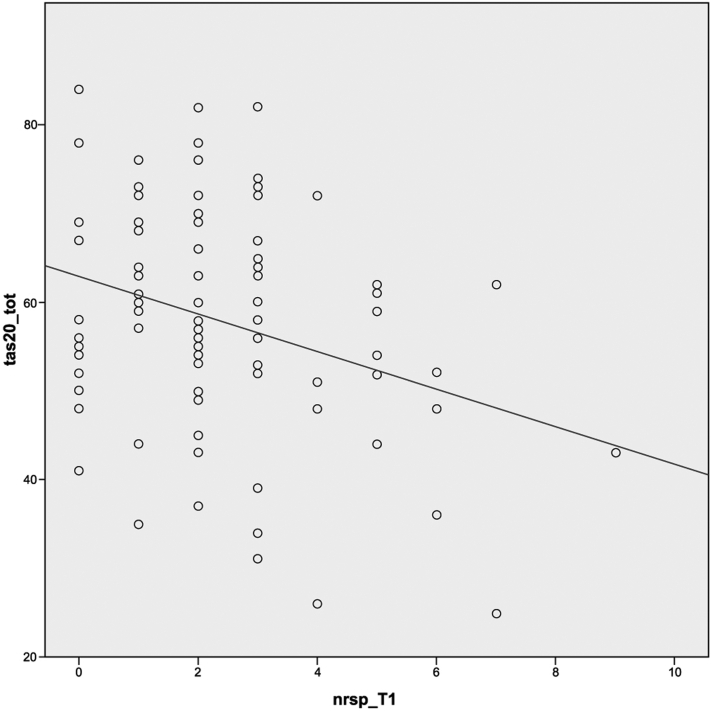
Correlation between Toronto Alexithymia Scale-20 item (TAS-20) and Pruritus Numerical Rating Scale for pruritus (NRSP) at T1. TAS-20 and NRSP are negatively related: higher values of alexithymia correspond to less intense values of itching (Spearman’s correlation: *r* = −0.230; *P* =.029).

A recent study concluded that the reduction in the impact on QoL for patients with AD in the first months of therapy with dupilumab correlates with the control of itching.^5^ Probably, because patients with alexithymia had a greater severity of the disease at the baseline than patients without alexithymia (EASI = 30.85 vs 23.50), treatment with anti–IL-4Rα for 16 weeks was most effective for them in reducing itching, and this positively affected their QoL. Further studies are needed to confirm these data.

In summary, to our knowledge, our study for the first time shows that the treatment with anti–IL-4Rα for AD reduces the prevalence of psychological burden in both patients with alexithymia and without alexithymia. In addition, in patients with alexithymia, anti–IL-4Rα improves clinical symptoms of AD, such as itching, and QoL more than in patients without alexithymia.

## Conflict of interest

None disclosed.
